# Frequency Following Response and Speech Recognition Benefit for Combining a Cochlear Implant and Contralateral Hearing Aid

**DOI:** 10.1177/2331216520902001

**Published:** 2020-01-31

**Authors:** David M. Kessler, Saradha Ananthakrishnan, Spencer B. Smith, Kristen D’Onofrio, René H. Gifford

**Affiliations:** 1 Department of Hearing and Speech Sciences, Vanderbilt University Medical Center, Nashville, TN, USA; 2 Department of Speech-Language Pathology & Audiology, Towson University, MD, USA; 3 Department of Communication Sciences and Disorders, The University of Texas at Austin, TX, USA; 4 Department of Otolaryngology, Vanderbilt University Medical Center, Nashville, TN, USA

**Keywords:** cochlear implants, hearing aids, bimodal hearing, bimodal benefit, electrophysiology

## Abstract

Multiple studies have shown significant speech recognition benefit when acoustic hearing is combined with a cochlear implant (CI) for a bimodal hearing configuration. However, this benefit varies greatly between individuals. There are few clinical measures correlated with bimodal benefit and those correlations are driven by extreme values prohibiting data-driven, clinical counseling. This study evaluated the relationship between neural representation of fundamental frequency (F0) and temporal fine structure via the frequency following response (FFR) in the nonimplanted ear as well as spectral and temporal resolution of the nonimplanted ear and bimodal benefit for speech recognition in quiet and noise. Participants included 14 unilateral CI users who wore a hearing aid (HA) in the nonimplanted ear. Testing included speech recognition in quiet and in noise with the HA-alone, CI-alone, and in the bimodal condition (i.e., CI + HA), measures of spectral and temporal resolution in the nonimplanted ear, and FFR recording for a 170-ms/da/stimulus in the nonimplanted ear. Even after controlling for four-frequency pure-tone average, there was a significant correlation (*r* = .83) between FFR F0 amplitude in the nonimplanted ear and bimodal benefit. Other measures of auditory function of the nonimplanted ear were not significantly correlated with bimodal benefit. The FFR holds potential as an objective tool that may allow data-driven counseling regarding expected benefit from the nonimplanted ear. It is possible that this information may eventually be used for clinical decision-making, particularly in difficult-to-test populations such as young children, regarding effectiveness of bimodal hearing versus bilateral CI candidacy.

## Introduction

Most unilateral cochlear implant (CI) recipients receive significant speech recognition benefit in quiet and in noise when acoustic hearing is added in the nonimplanted ear via a hearing aid (HA), also termed bimodal benefit (e.g., Dorman & Gifford, 2010; Dunn, Tyler, & Witt, 2005; Gifford, Dorman, McKarns, & Spahr, 2007; Sheffield & Gifford, 2014; Sheffield, Simha, Jahn, & Gifford, 2016; Zhang, Dorman, & Spahr, 2010). Yet, this bimodal benefit varies greatly between individuals, with some patients even experiencing a decrement in speech recognition performance with the addition of the contralateral HA (Gifford & Dorman, 2019; Mok, Grayden, Dowell, & Lawrence, 2006; Neuman et al., 2017; Zhang et al., 2010).

A number of studies have investigated this large variability in bimodal benefit by examining auditory characteristics of the nonimplanted ear and bimodal benefit with mixed results (Blamey et al., 2015; Gantz et al., 2009; Gifford, Dorman, Spahr, & Bacon, 2007; Illg, Bojanowicz, Lesinski-Schiedat, Lenarz, & Bu, 2014; Zhang, Spahr, Dorman, & Saoji, 2013). Audiometric thresholds have been found to only weakly correlate with bimodal benefit. Furthermore, this relationship is largely driven by patients with audiometric thresholds on the extreme ends of the hearing function (Blamey et al., 2015; Gantz et al., 2009; Illg et al., 2014; Marsh et al., 1993; Zhang et al., 2013). That is, patients with audiometric thresholds in the range of normal hearing (NH) sensitivity or mild hearing loss generally receive significant bimodal benefit and those with profound levels of hearing loss generally receive little-to-no bimodal benefit. However, this relationship for patients with moderate-to-severe hearing losses is less clear, and as such, it remains difficult to predict how much benefit one may receive. Thus, audiometric thresholds alone are unable to predict the presence or extent of bimodal benefit. Other aspects of auditory function such as temporal resolution measured via amplitude modulation detection thresholds, frequency selectivity quantified by auditory filter shapes at 500 Hz, and nonlinear cochlear processing measured via masked thresholds in the presence of positive and negative Schroeder phase harmonic complexes have been found to be equally unsuccessful in predicting acoustic benefit in listeners who met the preimplant criteria for combined electric and acoustic stimulation (Gifford, Dorman, Spahr, et al., 2007).

Zhang et al. (2013) sought to investigate the relationship between spectral resolution and audiometric thresholds of the nonimplanted ear and bimodal benefit in 22 bimodal patients. The authors found a significant relationship between audiometric thresholds and bimodal benefit across all participants. However, they noted that audiometric thresholds were not correlated with bimodal benefit within a group of participants with mild-to-moderate audiometric thresholds nor a group of participants with severe to profound audiometric thresholds. This supports previous findings that this relationship is largely driven by the extreme ends of the hearing function and highlights the limitations of audiometric thresholds for predicting bimodal benefit. They also reported a significant positive correlation (*r* = .895) between acoustic spectral modulation detection (SMD) and speech recognition benefit with access to acoustic hearing in the nonimplanted ear (i.e., bimodal benefit). Despite this correlation, it was unclear whether this laboratory-based measure could be clinically translated and whether the correlation would hold with much larger and more diverse populations of bimodal listeners.

It is evident from the studies reviewed earlier that we currently lack strong clinically available measures that explain variability in bimodal benefit, rendering clinical decisions regarding ear of implantation, and bilateral versus unilateral implantation not only challenging, but non-evidence-based. Furthermore, the exact cues driving bimodal benefit are unknown, prohibiting development and implementation of a clinical test for the nonimplanted ear. It is, however, understood that both spectral and temporal cues affect bimodal benefit. Proposed explanations for differences in speech recognition for bimodal listeners relate to acoustic processing of spectral cues such as formant frequency (F0) and low-frequency consonant cues (e.g., nasal and voicing cues) as well as temporal cues including periodicity and temporal fine structure (TFS). General convention holds that the temporal descriptions of sound characteristics are most commonly referencing temporal envelope, which describes the relatively slow changes in amplitude over time (Moore, 2008; Rosen, 1992). Temporal envelope provides cues to both manner and voicing (Rosen, 1992). Periodicity refers to the temporal representation of the glottal pulse or F0, often referred to as voice pitch. Periodicity is generally used as a cue to determine voicing or to aid suprasegmental representation of stress and intonation (Rosen, 1992). TFS of an acoustic stimulus refers to the rapid oscillation of sound waves with a rate close to the center frequency of the filter band which represents place of articulation and vocal quality (Rosen, 1992). It is believed that temporal representation of periodicity and TFS (and to a lesser degree temporal envelope) are represented in the auditory pathway via synchronous firing to the phase of the stimulus (i.e., phase locking). Although periodicity and TFS are often referenced in the temporal domain, both can also be analyzed in the spectral domain and contribute toward the processing of spectral cues. Furthermore, ability to use these cues may play an important role in F0 discrimination (Moore, Glasberg, Flanagan, & Adams, 2006; Schvartz-Leyzac & Chatterjee, 2015), listening in the dips of a fluctuating masker (Hopkins & Moore, 2009), sound source segregation (Assmann, 1996; Hong & Turner, 2009; Meddis & Hewitt, 1992; Qin & Oxenham, 2005; Summers & Leek, 1998; Vliegen, Moore, & Oxenham, 1999; Vliegen & Oxenham, 1999), and speech perception (Lorenzi, Gilbert, Carn, Garnier, & Moore, 2006; Sheft, Ardoint, & Lorenzi, 2008).

The primary difference between the two most prominent theories of bimodal benefit differs in terms of which temporal and spectral cues are considered the driving contributors. The theory of segregation posits that F0 is used to *segregate* the signal from the background noise. Although multiple studies have shown the importance of access to F0 information (Brown & Bacon, 2009a, 2009b; Chang, Bai, & Zeng, 2006; Kong, Stickney, & Zeng, 2005; Qin & Oxenham, 2006), others have argued that perhaps F0 is not the principal underlying cue used for bimodal benefit (Kong & Carlyon, 2007; Li & Loizou, 2008; Sheffield & Gifford, 2014). The theory of glimpsing proposes that bimodal listeners use voicing and TFS cues to *glimpse* the signal during spectrotemporal dips in the background noise. These cues allow the listener to know when the target speech is present and selectively focus on the target.

Further research by Sheffield and Gifford (2014) supported this idea of glimpsing, finding that bimodal benefit was comparable between equivalent low-pass and pass-band bandwidths (e.g., <250 Hz and 250–500 Hz bandwidths). This suggests that bimodal users can receive speech recognition benefit even without direct access to F0. However, segregation may still be taking place in this study. Lower frequencies, including the F0, may be resolved and extracted by the peripheral auditory system. That is, F0 information can still be extracted from the temporal information produced by unresolved F0 harmonics. It is therefore possible that the participants were able to use unresolved F0 harmonics to use voice pitch as a means to segregate target from distractor.

In addition to F0 cues from both resolved and unresolved components, voicing and phonetic cues as well as formant frequency information (e.g., F1) are also likely used to improve speech recognition performance. As such, there has been recent interest in using the frequency following response (FFR) as an objective tool to quantify F0 and F1 spectrotemporal processing in listeners with hearing loss given the importance of these cues for speech understanding (Ananthakrishnan, Krishnan, & Bartlett, 2016; Anderson, Parbery-Clark, White-Schwoch, Drehobl, & Kraus, 2013; Anderson, White-Schwoch, Choi, Kraus, & Peelle, 2013).

The human FFR is an auditory-evoked potential that reflects the synchronous neural activity originating in the auditory brainstem. However, it should be noted that recent evidence suggests that the FFR may additionally have a cortical contribution (Coffey, Herholz, Chepesiuk, Baillet, & Zatorre, 2016; Coffey, Musacchia, & Zatorre, 2017) though these contributions are likely weak (Bharadwaj et al., 2019; Bidelman, 2018) and unnecessary for FFR generation (White-Schwoch, Anderson, Krizman, Nicol, & Kraus, 2019). Unlike other electrophysiological measures such as the auditory brainstem response (ABR), the FFR is unique in that it accurately represents auditory characteristics of the stimulus, including temporal and spectral properties below ∼1500 Hz. Adding brainstem neural responses to stimuli presented in rarefaction and condensation polarities enhances the neural response to F0 and is called the envelope following response (EFR) or the FFR envelope. Conversely, subtraction of the brainstem neural responses to stimuli presented in rarefaction and condensation polarities provides the brainstem response reflecting phase locking to the harmonics, called the FFR to the TFS or the spectral FFR (Aiken & Picton, 2008). The FFR waveform therefore contains envelope, periodicity, and TFS of complex sounds. Thus, investigation of the FFR holds promise as an objective measurement of early sound processing in the auditory pathway that is unaffected by sleep.

A variety of studies have found a relationship between FFR spectral amplitudes and speech recognition in noise for participants with NH. This has been observed in adults ranging in age from 21 to 30 years (Song, Skoe, Banai, & Kraus, 2011) and 60 to 73 years (Anderson, Parbery-Clark, Yi, & Kraus, 2011), as well as in children aged 8 to 14 years (Anderson, Skoe, Chandrasekaran, & Kraus, 2010; Anderson, Skoe, Chandrasekaran, Zecker, & Kraus, 2010; Chandrasekaran, Hornickel, Skoe, Nicol, & Kraus, 2009; Hornickel, Chandrasekaran, Zecker, & Kraus, 2011; Hornickel, Skoe, Nicol, Zecker, & Kraus, 2009).

Results have been mixed in studies investigating the relationship between FFR and other behavioral measures in participants with NH. Clinard, Tremblay, and Krishnan (2010) found no relationship between FFR phase coherence or average spectral amplitude and frequency difference limens in 32 adults with NH. Conversely, other groups demonstrated a relationship between frequency discrimination and FFR neural pitch salience (i.e., FFR spectral magnitude within a specified frequency band) and FFR synchronization strength (Krishnan, Bidelman, Smalt, Ananthakrishnan, & Gandour, 2012; Marmel et al., 2013; Smalt, Krishnan, Bidelman, Ananthakrishnan, & Gandour, 2012). Bidelman, Gandour, and Krishnan (2011) demonstrated a relationship between FFR F0 magnitude and frequency discrimination in NH musicians, but not in a group of NH Mandarin speakers or nonmusicians, attributed to experience dependent neural plasticity. The differences across studies may be the result of diverse factors including, but not limited to, differences in FFR stimuli or methods of quantifying the FFR. Yet overall, these studies highlight the need for more research on the FFR and behavioral tasks of pitch and speech perception.

Much less is known about FFR in populations with sensorineural hearing loss. Extensive research has investigated the auditory steady state response (ASSR), a subcategory of the EFR (Dimitrijevic et al., 2016), in individuals with and without hearing loss (for review, see Picton, John, Dimitrijevic, & Purcell, 2003). However, unlike the FFR which provides TFS representation information, these studies often use tonal stimuli modulated in amplitude and frequency and do not assess TFS representation. Of the studies that exist, the majority of current evidence suggests that FFRs of individuals with hearing loss show differences compared to NH listeners in terms of a relative TFS deficit via enhanced FFR envelope magnitudes (Anderson, Parbery-Clark, et al., 2013) and reduced TFS magnitudes (Ananthakrishnan et al., 2016; Anderson, White-Schwoch, et al., 2013). This pattern of envelope enhancement and TFS degradation in speech-evoked FFRs is consistent with cochlear filter broadening secondary to sensorineural hearing loss. As cochlear filters broaden, particularly in channels tuned to higher acoustic frequencies, more harmonics of the speech stimulus are likely to fall within them (Gockel, Carlyon, Mehta, & Plack, 2011; Shinn-Cunningham, Ruggles, & Bharadwaj, 2013; Zhu, Bharadwaj, Xia, & Shinn-Cunningham, 2013). These harmonics interact and generate strong F0 periodicity within the cochlear filters, even if harmonic energy is at a relatively low sensation level (SL) due to hearing loss. Thus, the FFR envelope is not a measure of residual apical cochlear health at the *F0 place* but a measure of combined neural phase locking across all cochlear filters. The systematic impact of various degrees and configurations of hearing loss on FFR F0 energy recorded from the scalp is currently unclear.

Results are mixed regarding whether individuals with hearing loss also appear to have poorer neural synchrony than listeners with NH (Marmel et al., 2013; Plyler & Ananthanarayan, 2001). Furthermore, very few studies effectively control for age which has been associated with reduced FFR magnitude and phase coherence (Anderson, Parbery-Clark, et al., 2013; Bones & Plack, 2015; Clinard et al., 2010). To date, no published studies have evaluated the FFR in CI recipients who have aidable acoustic hearing in the nonimplanted ear, though poster presentation by D’Onofrio et al. (2018) showed a significant relationship between FFR F0 amplitude (170-ms/da/) in the nonimplanted ear and bimodal benefit for musical emotion perception.

During the CI evaluation process, patients are asked to consider potentially sacrificing some degree of acoustic hearing in one or both ears in exchange for a CI. To make a truly informed decision, patients should be provided with a reasonable prediction about how each ear might contribute to post-CI hearing and speech understanding; however, there are no available clinical measures that can reliably predict postoperative performance, guide the ear selection process, or clinically distinguish bimodal and bilateral CI candidates. As stated earlier, many listeners with acoustic hearing receive little-to-no bimodal benefit from ears that were labeled the *better hearing ear* during the preoperative evaluation (e.g., Gifford & Dorman, 2019; Neuman et al., 2017). Consequently, the decision about which ear to implant is often left to convenience, patient and surgeon preference, or even the proverbial coin flip. Thus, the purpose of this study is to replicate and expand on previous studies that have investigated the relationship between bimodal benefit and various aspects of auditory function in the nonimplanted ear. This study addresses the following research questions and associated hypotheses:

1. Does spectral resolution in the nonimplanted ear significantly relate to bimodal benefit? Based on findings by Zhang et al. (2013), we hypothesized that SMD performance in the nonimplanted ear would be significantly correlated with bimodal benefit. That is, as SMD improved (i.e., better spectral resolution), bimodal benefit would increase. We also hypothesized that a more specified measure of spectral resolution, psychophysical tuning curves (PTCs), would not be significantly related to bimodal benefit (Gifford, Dorman, Spahr, et al., 2007). Whereas PTCs assess spectral resolution at one place on the cochlear array, SMD can assess spectral resolution at one or several points along the cochlear array where the flat spectrum noise stimulus is audible. Thus, it is hypothesized that SMD performance, rather than PTC results, will be related to bimodal benefit because it is better able to quantify spectral resolution across the useable frequency range.
2. Is temporal resolution in the nonimplanted ear significantly related to bimodal benefit? We hypothesized that temporal envelope resolution in the nonimplanted ear would not be significantly correlated with bimodal benefit (Gifford, Dorman, Spahr, et al., 2007).
3. What is the relationship between FFR amplitude in the nonimplanted ear and bimodal benefit? Given that FFR spectral amplitude, particularly F0 spectral amplitude, has been shown to strongly correlate with speech recognition in noise for a variety of populations with NH (Anderson et al., 2011; Parbery-Clark, Skoe, & Kraus, 2009; Song et al., 2011), we hypothesized that FFR envelope spectrum amplitude at F0 and TFS spectrum amplitude at F1 would significantly correlate with bimodal benefit. Furthermore, we hypothesized that FFR-derived measures of TFS would be significantly correlated with bimodal benefit accounting for considerably more variance in bimodal benefit than accounted for by audiometric thresholds and spectral resolution.

## Methods

### Participants

All study procedures were approved by the Vanderbilt University Institutional Review Board (IRB # 171526), and all participants provided written informed consent prior to participation in the study. Participants for this study included 14 adults (11 women and 3 men) with sensorineural hearing loss who were unilaterally implanted with a current generation CI and wore a HA in the nonimplanted ear. At the time of testing, participants had a mean age of 57.8 years (*SD* = 15.7 years) with a range of 25.0 to 79.4 years of age. Across 14 participants, 6 were implanted with Advanced Bionics (AB), 3 with MED-EL, and 5 with Cochlear Ltd. devices. All but two participants self-identified as full-time HA users prior to completion of this study. Study inclusion criteria included at least 6 months of CI experience as well as audiometric thresholds ≤ 80 dB HL at 250 Hz in the nonimplanted ear. Table 1 provides a summary of participant demographic information including gender, age at testing, ear implanted, implant manufacturer, electrode type, duration of implant experience, aided speech intelligibility index (SII) in the nonimplanted ear, and full-time HA status. The Threshold Equalization Noise (TEN) HL Test (Moore, Glasberg, & Stone, 2004) was completed in the nonimplanted to test for the presence of cochlear dead regions (a region of the basilar membrane with few or no functioning inner hair cells). The test allows for measurements at 500, 750, 1000, 1500, 2000, 3000, and 4000 Hz. Per the test’s guidelines, a dead region was defined at a frequency where the masked threshold was 10 dB or higher than the unmasked threshold and the noise level.

**Table 1.** Demographic Information.

Subject number	Age at testing (years)	Ear implanted	Gender	Implant manufacturer	Device, electrode type	Years of CI use	Aided SII at 60 dB SPL	Full-time hearing aid user?
1	54.51	Left	Female	MED-EL	Concert, Standard	3.91	NA	Yes
2	70.31	Right	Female	AB	HiRes Ultra, mid-scala	1.20	29	Yes
3	62.17	Right	Female	Cochlear	CI24RE, L24	1.00	21	Yes
4	40.33	Left	Female	Cochlear	Profile, CI532	0.73	16	No
5	74.12	Left	Female	MED-EL	Synchrony, FLEX28	0.78	34	Yes
6	79.28	Left	Male	Cochlear	CI24RE, contour advance	5.95	11	Yes
7	46.65	Right	Female	AB	HiRes 90K Advantage, Mid-Scala	3.10	34	Yes
8	63.87	Left	Female	MED-EL	Synchrony, FLEX24	1.00	23	No
9	36.10	Right	Female	AB	HiRes Ultra, Mid-Scala	0.93	37	Yes
10	64.64	Right	Male	AB	HiRes 90K Advantage, 1j	5.76	48	Yes
11^a^	56.87	Left	Female	AB	HiRes 90K Advantage, Mid-Scala	4.66	30	Yes
12^a^	79.44	Left	Male	Cochlear	Profile, CI512	0.86	74	Yes
13	25.01	Right	Female	Cochlear	CI24RE, contour advance	10.60	53	Yes
14	55.59	Right	Female	AB	HiRes Ultra, Mid-Scala	1.45	38	Yes

*Note*. Age at testing, ear implanted, gender, implant manufacturer, electrode type, years of CI use, aided SII in the nonimplanted ear, and full-time HA user at the time of testing. NA = not applicable; AB = Advanced Bionics.

^a^Participant’s FFR data were not included due to experimenter error during stimulus presentation.

### Procedure

#### Audiometric thresholds and device verification

**Figure 1.**
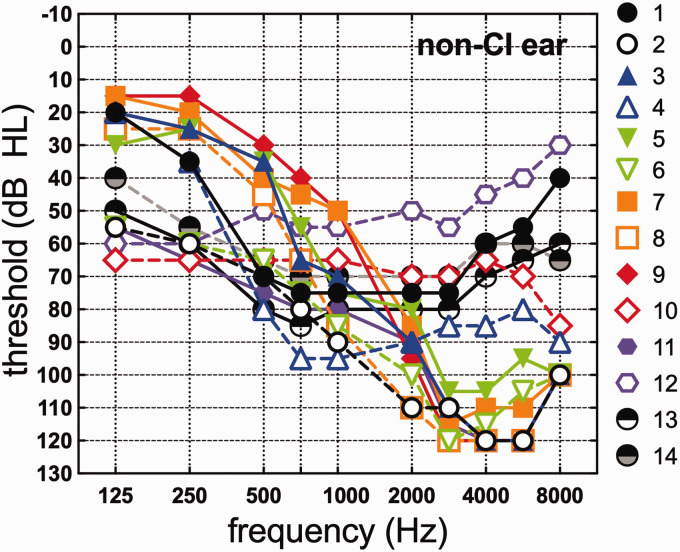
Audiometric thresholds (dB HL) in the nonimplanted ear from 125 to 8000 Hz for each participant.

All participants received a hearing evaluation to ensure they met study criteria. Audiometric thresholds were measured at octave and interoctave frequencies from 125 to 8000 Hz in the non-CI ear using ER-3A insert earphones and standard audiometry methods. Figure 1 displays air conduction audiometric thresholds from 125 to 8000 Hz in the nonimplanted ear for the 14 participants. In cases where no measurable threshold could be obtained at the limits of the audiometer, 5 dB was added to the no response threshold so as not to exclude data. The lowest frequency at which this occurred was 2000 Hz. The average low-frequency threshold for 125 to 750 Hz was 51.52 dB HL (*SD* = 14.65).

A real-ear-to-coupler difference was measured in the participant’s nonimplanted ear and was used for HA verification in the coupler. The participant’s HA was verified via an Audioscan RM500SL system test box using NAL-NL2 prescriptive targets (Keidser, Dillon, Flax, Ching, & Brewer, 2011). Participants were fit with a lab HA (ReSound Enzo Linx2) using a comply tip if their personal HA did not amplify within 5 dB of NAL-NL2 targets and could not be reprogrammed due to locked HA firmware. Aided SII was measured to provide quantification of audibility of speech using the HA (shown in Table 1). Aided CI thresholds were also measured in the sound booth using frequency modulated, pulsed tones from 250 to 6000 Hz including all interoctave frequencies. Aided detection thresholds were between 20- and 30-dB HL at all tested frequencies for all participants.

#### Speech recognition

Speech recognition was measured using guidelines recommended by the minimum speech test battery (Minimum Speech Test Battery, 2011) for adult CI users. This testing is commonly used in the audiology clinic for CI performance monitoring. Speech stimuli were presented at 60 dBA in a single-walled sound-treated booth via a speaker positioned at a 0° azimuth 1 m from the head of the participant. All participants were tested in three amplification configurations: (a) HA-only, (b) CI-only, and (c) bimodal (CI + contralateral HA). Participants wore a lapel microphone and were visible by the test administer on an LCD monitor in the control room via an in-booth video camera to aid in test scoring.

Speech recognition was assessed using consonant–nucleus–consonant (CNC) words (Peterson & Lehiste, 1962) in quiet as well as AzBio sentences (Spahr et al., 2012) in the presence of a 20-talker babble noise at a signal-to-noise ratio (SNR) of +5 dB. For CNC word recognition, participants were asked to repeat a one-syllable word that was preceded by the carrier phrase *ready* (e.g., *ready, duck*). Performance was scored in percentage of correct words. For AzBio sentences at +5 dB SNR, the speech stimuli and the noise were colocated from the speaker placed at 0 degrees (S_0_N_0_). Participants were encouraged to repeat as much of the sentence as they heard and to guess if they were not sure. Performance was scored as the percentage of correct words.

In addition to clinical measures of speech recognition, participants also completed sentence recognition testing presented in the Revitronix R-SPACE™ sound simulation system, which uses a prerecorded restaurant noise to simulate a real-world listening environment. Here, the listener is surrounded by a circular array of eight loudspeakers placed at 45° intervals located 24 in. from the center of the participant’s head. R-SPACE™ system design and methods for recording restaurant environmental noise have been discussed in previous studies (Compton-Conley, Neuman, Killion, & Levitt, 2004; Revit, Killion, & Compton-Conley, 2007). AzBio sentences were presented at a level of 67 dBA at an SNR of +5 dB from a speaker at an azimuth of zero degrees with the restaurant noise presented from the remaining seven speakers (S_0_N_45–315_). These speech and noise levels were chosen to be consistent with typical levels reported for upscale restaurants which have more favorable listening conditions (Farber & Wang, 2017; Lebo et al., 1994). As before, participants were tested in three listening configurations: (a) HA-only, (b) CI-only, and (c) bimodal (CI + contralateral HA). Raw scores for all three speech recognition measures were converted to rationalized arcsine units (rau; Studebaker, 1985) which were used for all subsequent analyses. Converting speech recognition proportion scores to rau allows for more appropriate statistical analyses and attempts to minimize floor and ceiling effects.

#### Bimodal benefit

In this study, bimodal benefit was quantified in two ways. The first method was calculated by subtracting speech recognition performance with the CI-alone from performance in the bimodal condition, hereafter referred to as acoustic benefit. For example, a participant who received a score of 50 rau in the CI-alone condition and 75 rau in the bimodal condition would have an acoustic benefit of 25 rau. Although this method makes intuitive sense, it does not account for differences in CI-alone performance across participants and thus is unable to fully control for floor and ceiling effects, even with the transformed scores. This has the potential of minimizing the amount of observed bimodal benefit.

To account for CI-alone performance, a second method for calculating bimodal benefit was used, hereafter referred to as normalized acoustic benefit. Normalized acoustic benefit divides acoustic benefit by the amount of possible rau improvement multiplied by 100. It should be noted that the maximum possible rau improvement depends on the speech recognition stimuli as the transform considers the number of items in the test. For CNC words and AzBio sentences, the maximum rau value is 116.47 and 119.11, respectively. To use the previous example, if the patient had a CNC CI-alone score equal to 50 rau and a bimodal score equal to 75 rau, normalized acoustic benefit would be equal to 37.6%. In other words, the patient achieved 37.6% of the total possible benefit (i.e., 25 of the total 66.47 rau possible improvement before hitting ceiling). The equation for normalized acoustic benefit is as follows:

$$\begin{array}{l} \text{For}\;\text{CNC}\;\text{words}:\text{1}00\times (\left(\text{bimodal}-\text{CI}-\text{Alone}\right)\\ \;/\left(\text{116}.\text{47}-\text{CI}-\text{Alone}\right)) \end{array}$$

$$\begin{array}{l} \text{For}\;\text{AzBio}\;\text{sentences}:\text{1}00\times ((\text{bimodal}-\text{CI}-\text{Alone})\\ \;/(\text{119}.\text{11}-\text{CI}-\text{Alone})) \end{array}$$

In the scenario that speech recognition decreased with the addition of a HA to the nonimplanted ear (i.e., CI-alone score > bimodal score), the equation was reworked to divide the acoustic benefit by the amount of possible rau decrement. As before, the maximum amount of possible rau decrement depends on the speech recognition stimulus. For CNC words and AzBio sentences, the minimum rau value is −16.47 and −19.11, respectively. As all CI-alone scores were greater than 0, the equation was able to be reworked as follows:

$$\begin{array}{l} \text{For}\;\text{CNC}\;\text{words}:\text{1}00\times ((\text{bimodal}-\text{CI}-\text{Alone})\\ \;/(\text{CI}-\text{Alone}+\text{16}.\text{47})) \end{array}$$

$$\begin{array}{l} \text{For}\;\text{AzBio}\;\text{sentences}:\text{1}00\times ((\text{bimodal}-\text{CI}-\text{Alone})\\ \;/(\text{CI}-\text{Alone}+\text{19}.\text{11})) \end{array}$$

These equations result in a score from −100% to 100% indicating a decrement in performance in the bimodal condition to the minimum rau value and improvement in performance in the bimodal condition to the maximum rau value, respectively. This method of calculation is described further with raw speech recognition scores in the publication by Zhang et al. (2013).

#### Spectral resolution

Participants completed two measures of spectral resolution in the nonimplanted ear: (a) quick spectral modulation detection (QSMD) task and (b) a fast method of measuring PTCs.

The QSMD task (Gifford, Hedley-Williams, & Spahr, 2014) consisted of a three-interval forced-choice task with two intervals containing flat spectrum noise (125–5600 Hz) and the remaining interval was frequency modulated at a constant modulation rate of 1.0 cycle per octave. The QSMD was designed for presentation to acoustic hearing ears (Holder, Levin, & Gifford, 2018) and uses a method of constant stimuli including modulation depths ranging from 4 to 22 dB in 2-dB steps. Participants completed 60 trials for a total of 6 trials at each of the 10 modulation depths. The stimuli were presented to the nonimplanted ear via ER-3A insert earphones at a comfortable loudness level as reported by the participant. Comfortable loudness level as determined by the participant ranged from 88 to 108 dB SPL (mean = 101.79 dB SPL, *SD* = 6). Individual SLs of the QSMD stimulus were measured using each participant’s pure-tone average (PTA) at 500, 1000, and 2000 Hz converted to dB SPL as reference. On average, the SL of the stimulus was 23.8 dB SL (*SD* = 10.2). Participants selected which interval sounded different than the other two by selecting the corresponding number on a touchscreen. Performance was scored as percent correct at each modulation depth constructing a psychometric function using a general linear model. Specifically, a logit link function was generated for each subject using the MATLAB statistics toolbox function glmfit. This psychometric function was used to extract a threshold (to the nearest dB) for the modulation depth corresponding to 70% correct.

The fast method for determining PTCs (Sęk, Alcántara, Moore, Kluk, & Wicher, 2005; Sęk & Moore, 2012) was originally created with the purpose of quickly measuring frequency selectivity and identifying dead regions within the cochlea. For this task, the participant listened to a sinusoidal pulsed tone set to a level just above threshold presented to the nonimplanted ear via an ER-3A insert earphone. Each pulsed tone was presented with a duration of 500 ms with 20 ms rise and decay time windowed by a cosine gate function. Testing was completed with a 262 Hz and 440 Hz tone to measure the PTC at these frequencies for each participant. Low-frequency stimuli were selected to measure spectral resolution at a frequency range close to the F0 and F1 spectral characteristics of the FFR stimulus. In addition, these frequencies were selected prior to the recruitment of participants. As better audiometric thresholds are commonly seen for low frequencies in the nonimplanted ear of bimodal listeners, low-frequency stimuli provided the greatest chance for sufficient audibility for successful completion of the task by all participants.

Throughout the duration of testing (1 run =3 minutes), a narrowband noise masker was presented. For the 262-Hz tone, the noise had a center frequency which was swept from 131 Hz (*f*_min_) to 393 Hz (*f*_max_) with a bandwidth of 52 Hz. For the 440-Hz tone, the noise had a center frequency which was swept from 220 Hz (*f*_min_) to 660 Hz (*f*_max_) with a bandwidth of 88 Hz. A run was completed with the noise masker swept from *f*_min_ to *f*_max_ and another run was completed with the noise swept from *f*_max_ to *f*_min_ for a total of four runs (two runs per tested frequency). The participant was instructed to press and hold the space bar on a keyboard whenever she or he heard the tone and release the space bar when the tone was no longer audible. When the space bar was pressed, the narrowband noise increased in level at a rate of 2 dB/sec and decreased at the same rate when the space bar was released. At the completion of each test, Q10 (i.e., measurement of the PTC bandwidth) and an estimation of the PTC tip frequency were recorded. Q10 values were automatically measured in the SWPTC software using double regression and quadratic function measurements.

#### Temporal resolution

Amplitude modulation (AM) detection thresholds were measured in the nonimplanted ear using a three-interval forced choice task to acquire a psychophysical estimate of temporal envelope resolution. A flat spectrum noise carrier with a bandwidth of 125 to 5600 Hz was modulated using two different modulation frequencies: 4 Hz and 128 Hz. The stimulus was presented at 90 dB SPL via an ER-3A insert earphone. Using a method of constant stimuli, modulation index, 20log(m), ranged from 0 dB to −22 dB in 2-dB steps for each modulation frequency. Three trials were completed at each of the 12 modulation indices for a total of 36 trials per modulation frequency. Average percent correct across the three trials was calculated at each modulation index and plotted to generate a psychometric function using a general linear model. The psychometric function was created using a similar method as the earlier described QSMD analysis. Threshold was determined by the modulation index corresponding to 70% correct.

#### Frequency following response

FFR was measured in the nonimplanted ear using a 170-ms/da/stimulus (fundamental frequency (F0) = 100 Hz, first formant frequency (F1) = 700 Hz) presented using a magnetically shielded Etymotic ER-3C insert earphone in a sound-treated booth at a rate of 4.35 Hz. Stimuli were presented at a fixed intensity of 90 dB SPL. Each measurement included an average of 3,000 repetitions of the stimulus with artifact rejection set to ±31 µV. A high-pass filter was set to 1 Hz and a low-pass filter was set to 5000 Hz to allow for post hoc filtering. The stimulus was presented with alternating polarities to allow for the addition and subtraction of the responses measured in condensation and rarefaction polarities during analysis. Processing the data in this way provides access to neural encoding of specific features such as envelope (added) and TFS (subtracted; Aiken & Picton, 2008) The stimulus was generated and presented via an Intelligent Hearing System (IHS) Duet System (Smart EP, Miami FL, USA). Testing was completed using a vertical montage with a three Ag-AgCl electrode array (Cz active, Fpz ground, ipsilateral earlobe reference). Impedance criteria were set at ≤6 kilo-ohms for all electrode contacts. Participants sat in a reclining chair and were asked to sit in a relaxed position while staying awake. The CI in the opposite ear was inactive during FFR recording.

Each participant completed two runs resulting in two recorded FFRs for a total test duration of 30 minutes. The two FFRs were averaged together and post hoc filtered with a bandpass filter of 70 to 3000 Hz. The averaged recording was saved as an ASCII file and the steady state vowel portion of the FFR (60–180 ms) was transformed into the frequency domain using a fast Fourier transform (FFT) with 1-Hz resolution. The envelope of the FFR is enhanced by the addition of opposite polarities, whereas TFS is diminished. As the F0 of the stimulus was 100 Hz, the amplitude of the envelope spectrum at 100 Hz was analyzed for each participant. The alternate stimulus polarities were also subtracted, effectively enhancing the spectral components of the FFR and eliminating the FFR envelope (see Aiken & Picton, 2008) for a more detailed explanation of this procedure). The spectral amplitude of the subtracted polarities was recorded at F1 of the stimulus (i.e., 700 Hz). FFR data from two participants (Subjects 11 and 12) were not included in the following analyses due to experimenter error during FFR collection. Specifically, the FFR recordings for those participants were completed with an 80 dB SPL presentation instead of 90 dB SPL.

#### Questionnaire

Given that musical training and years of music experience is related to differences in the FFR (Bidelman et al., 2011; Bidelman, Weiss, Moreno, & Alain, 2014; Parbery-Clark, Anderson, Hittner, & Kraus, 2012; Strait, O’Connell, Parbery-Clark, & Kraus, 2013), participants completed the Ollen Musical Sophistication Index (OMSI; Ollen, 2006). The OMSI is a 10-question online survey that provides a score representing the probability that a music expert would categorize the respondent as *more musically sophisticated*. Scores over 500 indicate that the respondent has a greater than 50% likelihood of being classified as more musically sophisticated, and scores less than 500 indicate that the respondent has a less than 50% likelihood of being classified as more musically sophisticated and is therefore classified as *less musically sophisticated*.

## Results

### TEN Test

Results of the TEN test revealed a present cochlear dead region at one or more of the tested frequencies for six participants. Of these participants, two had a dead region at 750 Hz (Subjects 1 and 8), two at 1000 Hz (Subjects 4 and 8), four at 1500 Hz (Subjects 4, 6, 9, and 14), and two at 2000 Hz (Subjects 1 and 14).

### Speech Recognition

**Figure 2.**
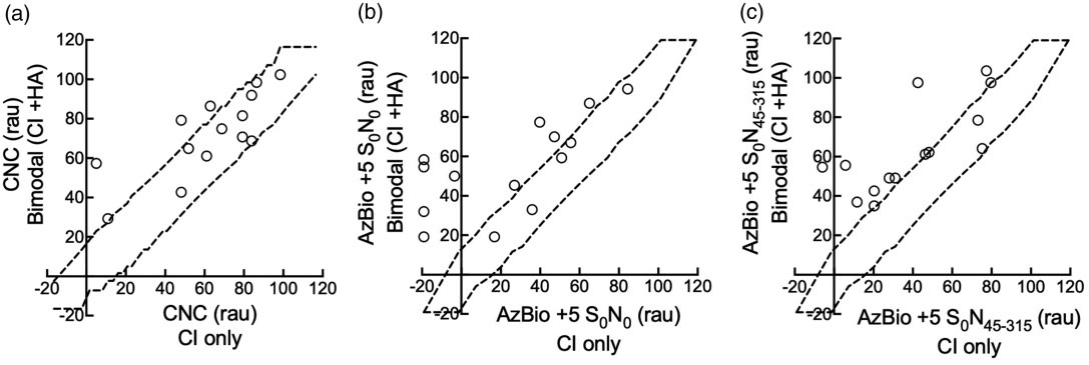
Speech recognition scores (rau) for CNC (a), AzBio sentences in +5 dB SNR (S_0_N_0_) (b), and AzBio sentences in +5 dB SNR (S_0_N_45–315_) (c) listening with the CI-only (*x*-axis) and in the bimodal condition (*y*-axis). See the main text for further details. CNC = consonant–nucleus–consonant; CI = cochlear implant; HA = hearing aid.

For CNC words, average performance in HA-alone, CI-alone, and bimodal listening conditions was 39.6 rau (*SD* = 20.2), 62.0 rau (*SD* = 27.6), and 72.1 rau (*SD* = 20.5), respectively. For AzBio sentences at +5 dB SNR (S_0_N_0_), average performance in the HA-alone, CI-alone, and bimodal listening conditions was 25.1 rau (*SD* = 26.6), 24.5 rau (*SD* = 35.3), and 54.8 rau (*SD* = 223.5), respectively. Finally, average performance for AzBio sentences at +5 dB SNR (S_0_N_45–315_) in the HA-alone, CI-alone, and bimodal listening conditions was 31.7 rau (*SD* = 25.3), 39.5 rau (*SD* = 27.4), and 63.4 rau (*SD* = 21.8), respectively. Individual speech recognition scores in the CI-only and the bimodal listening conditions for CNC words in quiet as well as AzBio sentences in +5 dB SNR (S_0_N_0_ and S_0_N_45–315_) are shown in Figure 2(a) to (c), respectively. For each figure, dotted lines indicate 95% confidence intervals for the difference test stimuli (Spahr et al., 2012; Thornton & Raffin, 1978). That is, points within the dotted lines indicate no significant difference between CI-only and bimodal scores, for a given listener. Points falling above the top dotted line indicate significantly higher scores in the bimodal condition compared to CI-only scores and points falling below the bottom line represent significantly higher scores in the CI-only condition compared to bimodal scores. No participants in the current sample showed significantly poorer performance in the bimodal condition compared to the CI-only condition. Participants either showed similar performance in the CI-only and bimodal conditions or significantly better performance in the bimodal condition compared to the CI-only condition. Repeated measures analysis of variance revealed a significant difference between CI-alone and bimodal scores for CNC words, *F*(1, 13) = 4.72, *p* = .049, ${\text{η}}_{\text{p}}^{\text{2}}$ = 0.27, AzBio sentences at +5 dB SNR (S_0_N_0_), *F*(1, 13) = 19.40, *p* < .0001, ${\text{η}}_{\text{p}}^{\text{2}}$ = 0.60), and for AzBio sentences at +5 dB SNR (S_0_N_45–315_), *F*(1, 13) = 21.10, *p* < .0001, ${\text{η}}_{\text{p}}^{\text{2}}$ = 0.62.

**Figure 3.**
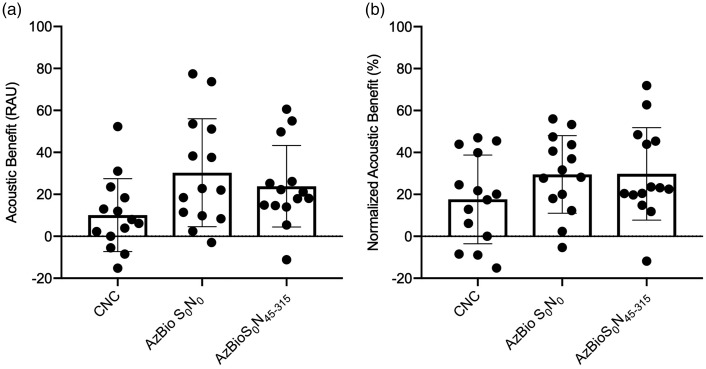
Average acoustic benefit (a) measured in rau and normalized acoustic benefit (b) measured in percent for CNC words in quiet, AzBio sentences at +5 dB SNR (S_0_N_0_ and S_0_N_45–315_). Error bars represent ± 1 standard deviation. CNC = consonant–nucleus–consonant.

As detailed in the Methods section, bimodal benefit was measured in two ways: acoustic benefit and normalized acoustic benefit. Figure 3(a) displays acoustic benefit, in rau, for CNC and AzBio at +5 dB SNR (S_0_N_0_ and S_0_N_45–315_). Average acoustic benefit was 10.1-, 30.3-, and 23.8-rau for CNC, AzBio +5 dB SNR (S_0_N_0_), and AzBio +5 dB SNR (S_0_N_45–315_), respectively. Figure 3(b) displays normalized acoustic benefit for CNC and AzBio at +5 dB SNR (S_0_N_0_ and S_0_N_45–315_). Average normalized benefit was 17.6%, 29.5%, and 29.8% for CNC, AzBio +5 dB SNR (S_0_N_0_), and AzBio +5 dB SNR (S_0_N_45–315_), respectively.

### Additional Correlations

Correlation analyses were completed comparing audiometric threshold at 125 Hz, four-frequency PTA (500, 1000, 2000, and 4000 Hz), aided SII, and age at testing with both acoustic benefit (Table 2) and normalized acoustic benefit (Table 3) for the three speech recognition measures. Although this was not a primary research question, aided SII and four-frequency PTA were significantly correlated (*r* = .82, *p* = .0006), as expected; however, aided SII was included in analysis as it provides a more precise measure of audibility to speech spectrum (McCreery et al., 2015). Since we completed 12 correlation analyses for each dependent variable (bimodal benefit and normalized bimodal benefit), we applied a Bonferroni correction for multiple comparisons which adjusts α to .004 for significance. There was no statistically significant correlation between any of the four independent variables and acoustic or normalized acoustic benefit after accounting for multiple comparisons. Although not reaching statistical significance, relationships between audiometric threshold at 125 Hz and bimodal benefit for AzBio +5 dB SNR (S_0_N_0_; acoustic and normalized acoustic benefit) as well as age at testing and bimodal benefit for AzBio +5 dB SNR (S_0_N_0_) exhibited strong correlation coefficients.

**Table 2.** Correlation Coefficients for Measures of Acoustic Benefit and Audiometric Threshold at 125 Hz, Four-Frequency PTA (0.5, 1, 2, and 4 kHz), Aided SII, and Age at Testing.

Measures	CNC	AzBio +5 dB SNR S_0_N_0_	AzBio +5 dB SNR S_0_N_45–315_
Audiometric threshold at 125 Hz	0.31 (*p* = .29)	0.69 (*p* = .006)	0.12 (*p* = .67)
Four-frequency PTA (0.5, 1, 2, and 4 kHz)	0.4 (*p* = .16)	0.01 (*p* = .99)	0.09 (*p* = .77)
Aided SII	0.25 (*p* = .40)	0.07 (*p* = .82)	0.01 (*p* = .98)
Age at testing	0.14 (*p* = .63)	0.50 (*p* = .07)	0.15 (*p* = .61)

CNC = consonant–nucleus–consonant; SII = speech intelligibility index; PTA = pure-tone average; SNR = signal-to-noise ratio.

**Table 3.** Correlation Coefficients for Measures of Normalized Acoustic Benefit and Audiometric Threshold at 125 Hz, Four-Frequency PTA (0.5, 1, 2, and 4 kHz), Aided SII, and Age at Testing.

Measures	CNC	AzBio +5 dB SNR S_0_N_0_	AzBio +5 dB SNR S_0_N_45–315_
Audiometric threshold at 125 Hz	0.06 (*p* = .84)	0.59 (*p* = .03)	0.11 (*p* = .7)
Four-frequency PTA (0.5, 1, 2, and 4 kHz)	0.35 (*p* = .22)	0.05 (*p* = .86)	0.10 (*p* = .72)
Aided SII	0.36 (*p* = .22)	0.11 (*p* = .71)	0.19 (*p* = .54)
Age at testing	0.20 (*p* = .50)	0.66 (*p* = .01)	0.23 (*p* = .43)

CNC = consonant–nucleus–consonant; SII = speech intelligibility index; PTA = pure-tone average; SNR = signal-to-noise ratio.

### Spectral Resolution and Bimodal Benefit

#### QSMD

Across all participants, the average acoustic QSMD threshold was 6.4 dB (*SD* = 3.9 dB). A correlation analysis was performed comparing QSMD threshold (in dB) in the nonimplanted ear and both acoustic and normalized acoustic bimodal benefit for CNC words, AzBio sentences at +5 dB SNR (S_0_N_0_), and AzBio sentences at +5 dB SNR (S_0_N_45–315_). Across all six correlations, there was no statistically significant correlation between QSMD thresholds and any measure of bimodal benefit. Correlations between QSMD threshold and bimodal benefit were as follows: normalized acoustic benefit for CNC words (*r* = .47, *p* = .09), normalized acoustic benefit for AzBio +5 (S_0_N_0_; *r* = .49, *p* = .08), normalized acoustic benefit for AzBio +5 (S_0_N_45–315_; *r* = .15, *p* = .61), acoustic benefit for CNC words (*r* = .39, *p* = .17), acoustic benefit for AzBio +5 (S_0_N_0_; *r* = .41, *p* = .15), and acoustic benefit for AzBio +5 (S_0_N_45–315_; *r* = .22, *p* = .44). Although none of the correlations reached statistical significance for an α of .05, according to Cohen’s classification (Cohen, 1988), many of the correlation coefficients would be consistent with a medium or moderate effect size and thus trending toward greater bimodal benefit with better spectral resolution.

#### Swept PTC

The sharpness (i.e., selectivity) of the auditory tuning curve in the nonimplanted ear at 262 Hz and 440 Hz was measured using the quick method for acquiring the PTC (Sęk et al., 2005; Sęk & Moore, 2012). Q10 was the metric used to indicate sharpness of the tuning curve. Lower numbers indicate broader tuning, whereas a higher Q10 value indicates more sharply tuned PTCs (i.e., better frequency selectivity). Q10 values derived from quadratic functions were used for analysis. In the case that a Q10 could not be measured by the SWPTC software (440 Hz stimulus, *n* = 1), the double regression Q10 value was used. Furthermore, when a participant’s tuning curve was so broad that a Q10 could not be recorded with a quadratic function or double regression, the participant was assigned a value of 0 for that frequency (262 Hz stimulus, *n* = 3; 440 Hz stimulus, *n* = 1). Mean Q10 value and center frequency for the 262 Hz stimulus was 1.54 (*SD* = 0.84) and 270.3 Hz (*SD* =21.96 Hz), respectively. For the 440 Hz stimulus, the mean Q10 value was 1.5 (*SD* = 0.75) and the average center frequency was 441.3 Hz (*SD* = 41.1 Hz).

Correlational analyses were completed to compare Q10 values at 262 and 440 Hz with acoustic and normalized acoustic benefit for CNC words and AzBio sentences at +5 dB SNR (S_0_N_0_ and S_0_N_45–315_) for a total of 12 correlations. There were no statistically significant correlations for any of the correlational analyses (*p* ≥ .05) with correlation coefficients ranging from .01 to .38. There was still no significant correlation between Q10 values (262 and 440 Hz) and acoustic or normalized acoustic benefit for any speech recognition measure (*p* ≥ .05, correlation coefficients ranging from .02 to .26) when participant Q10 data equal to 0 was excluded.

#### Temporal resolution and bimodal benefit

Temporal resolution, via sinusoidal AM detection, was quantified as the modulation index, between 0 and −22 dB, necessary to reach a performance level of 70% correct. Two runs were completed for each participant, one for each modulation frequency (i.e., 4 and 128 Hz). Four participants had sufficiently poor performance with a 128 Hz modulation frequency that their performance did not reach 70% correct even for 100% modulation (m = 1.0). Thresholds for these individuals were assigned as 0 dB (*n* = 4). Mean AM detection thresholds were 12.4 dB (*SD* = 4.2 dB) and 6.7 dB (*SD* = 6.0 dB) for 4 and 128 Hz, respectively.

Correlation analyses were completed for temporal modulation thresholds at both modulation frequencies and bimodal benefit, both acoustic and normalized acoustic benefit, for CNC and AzBio +5 dB SNR (S_0_N_0_ and S_0_N_45–315_). This resulted in a total of 12 correlational analyses. There were no statistically significant correlations between acoustic and normalized acoustic benefit for any speech recognition measure and temporal envelope resolution at either modulation frequency (*p* ≥ .05) with correlation coefficients ranging from .01 to .47.

Excluding the four participants’ data for whom an AM detection threshold of 0 dB was assigned at the 128 Hz modulation frequency, there was still no significant correlation between temporal resolution and either acoustic or normalized acoustic benefit for any of the speech recognition measures (*p* ≥ .05) with correlation coefficients ranging from .01 to .45.

#### FFR and bimodal benefit

**Figure 4.**
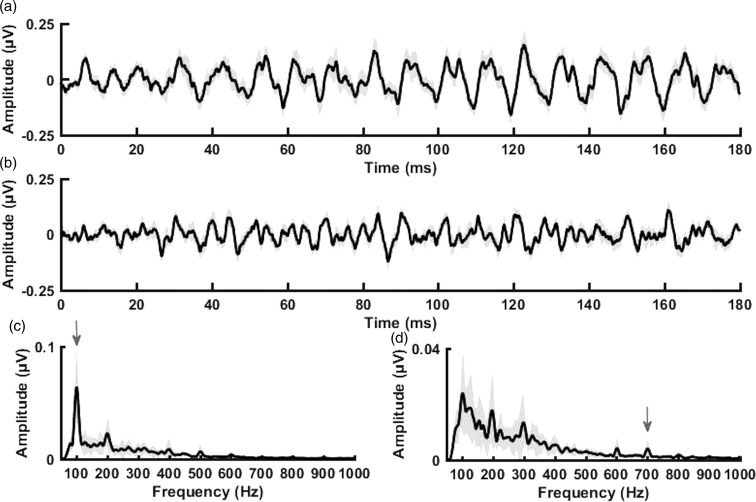
Grand average envelope FFR waveform (a), grand average fine structure FFR waveform (b), FFR envelope spectrum (c), and FFR fine structure spectrum (d). Note that the envelope and fine structure spectra were calculated over the steady state (60–180 ms) range of the response waveform. Shaded regions represent *SEM*. Arrows point to amplitude at F0 (100 Hz; c) and F1 (700 Hz; d).

Figure 4(a) and (b) displays the grand average envelope FFR waveform and the grand average fine structure FFR waveform for the 12 participants whose data could be used for analyses, respectively. Figure 4(c) and (d) displays the grand average envelope spectra and grand average fine structure spectra, respectively. The shading around the grand average waveforms and spectra represents ±1 standard error of the mean (*SEM*). The average FFR spectral envelope amplitude at F0 (i.e., 100 Hz) was equal to 0.08 µV (*SD* = 0.04) and the average FFR spectral fine structure amplitude at F1 (i.e., 700 Hz) was equal to 0.005 µV (*SD* = 0.004), shown by the arrows in Figure 4(c) and (d), respectively. Overall, the participants in this study had present but variable representation of F0 and poor neural representation of F1 (i.e., small fine structure spectral amplitudes at 700 Hz). To ensure that recorded spectral F0 and F1 amplitudes reflected true spectral neural representation and not part of the recording noise floor, FFR noise floors were estimated at frequencies of interest (i.e., F0 and F1) by calculating the FFT of the pre-stimulus interval (−20 to 0 ms). FFR response components were only considered present if their amplitudes were above the estimated noise floor determined using methods outlined by Russo, Nicol, Musacchia, and Kraus (2004). In this method, FFR amplitudes for F0 and F1 were divided by the FFR noise floors at F0 and F1, respectively. FFR responses were considered above the noise floor if quotients were greater than or equal to one. F0 and F1 FFR components were present in all 12 subjects using this criterion.

An additional analysis was completed to determine whether the presence or absence of a cochlear dead region as measured by the TEN test impacted FFR amplitude. Of the 12 participants with FFR data, 6 participants had at least one measurable cochlear dead region and 6 participants had no measurable cochlear dead region. An unpaired two-tailed *t* test assuming unequal variances showed no significant difference between groups of individuals with and without cochlear dead regions related to FFR F0 amplitude, *t*(10) = 0.3, *p* = .77, or FFR F1 amplitude, *t*(10) = 0.2, *p* = .84.

**Figure 5.**
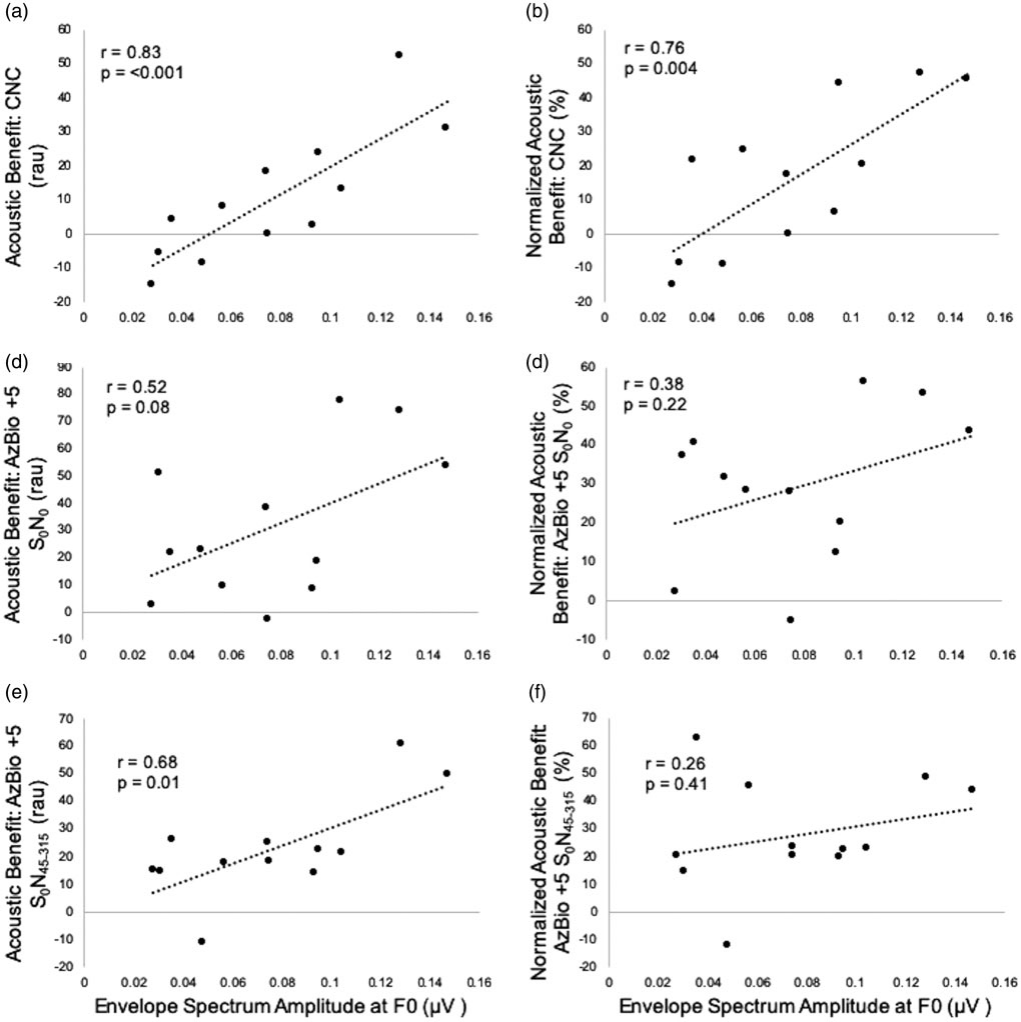
Acoustic benefit (left column) measured in rau and normalized acoustic benefit (right column) measured in percent for CNC words (a and b), AzBio sentences in +5 dB SNR (S_0_N_0_) (c and d), and AzBio sentences in +5 dB SNR (S_0_N_45–315_) (e and f) as a function of envelope spectrum amplitude of the frequency following response at the fundamental frequency (F0, 100 Hz). CNC = consonant–nucleus–consonant.

Correlation analyses were completed for spectral amplitudes of the FFR at the F0 of the stimulus (100 Hz) and at the first formant of the stimulus (700 Hz) and acoustic and normalized acoustic benefit for the three speech recognition tasks. For CNC words, F0 amplitude was significantly correlated with acoustic (*r* = .83, *p* < .001) and normalized acoustic benefit (*r* = .76, *p* = .004) as shown in Figure 5(a) and (b), respectively. That is, greater neural representation of F0 was associated with significantly higher acoustic and normalized acoustic benefit. There was no statistically significant relationship between FFR amplitude at F1 and acoustic or normalized acoustic benefit for CNC words (*p* ≥ .05), though this is likely due to the fact that F1 was poorly represented in the individual FFRs as a result of the magnitude of hearing loss at 700 Hz and above (Figure 1).

For AzBio sentences at +5 dB SNR (S_0_N_0_), there was no significant relationship between FFR F0 amplitude and acoustic benefit (*r* = .52, *p* = .08) or normalized acoustic benefit (*r* = .38, *p* = .22) displayed in Figure 5(c) and (d), respectively. As with CNC words, there was no relationship between FFR amplitude at F1 and acoustic or normalized acoustic benefit (*p* ≥ .05). For AzBio +5 dB SNR (S_0_N_45–315_), there was a significant positive correlation between FFR amplitude at F0 and acoustic benefit (*r* = .68, *p* = .01) but not for normalized acoustic benefit (*r* = .26, *p* = .41) as shown in Figure 5(e) and (f), respectively. Consistent with other measures, there was no significant correlation with FFR F1 amplitude and either metric of bimodal benefit (*p* ≥ .05).

A partial correlation was then computed between FFR F0 amplitude and bimodal benefit, controlling for four-frequency PTA. If four-frequency PTA was the principle determinant of bimodal benefit, the partial correlation between FFR F0 amplitude and bimodal benefit should not be significant. First considering acoustic benefit, FFR F0 amplitude was correlated with bimodal benefit for CNC words, *r*_(10)_ = .73, *p* = .01, even when controlling for four-frequency PTA and accounting for multiple comparisons via Bonferroni correction. For speech recognition in noise, the results of partial correlation analysis revealed that FFR F0 amplitude was not significantly correlated with bimodal benefit for AzBio +5 in the colocated condition (S_0_N_0_), *r*(10) = .49, *p* = .12, after controlling for four-frequency PTA. In the spatially separated condition (S_0_N_45–315_), FFR F0 amplitude was correlated with acoustic benefit, *r*(10) = .63, *p* = .04, even when controlling for four-frequency PTA; however, after accounting for multiple comparisons via Bonferroni correction, this correlation did not quite meet statistical significance.

Next considering normalized acoustic benefit, FFR F0 amplitude was correlated with bimodal benefit for CNC words, *r*(10) = .62, *p* = .04, even when controlling for four-frequency PTA; however, after accounting for multiple comparisons via Bonferroni correction, this correlation did not meet statistical significance. For speech recognition in noise, the results of partial correlation analysis revealed that FFR F0 amplitude was not significantly correlated with bimodal benefit for AzBio +5 in the colocated condition (S_0_N_0_), *r*(10) = .21, *p* = .53, or in the spatially separated condition (S_0_N_45–315_), *r*(10) = .06, *p* = .87, when controlling for four-frequency PTA.

In summary, FFR F0 amplitude was significantly correlated with bimodal benefit for CNC words in quiet and AzBio sentences at +5 dB SNR (S_0_N_45–315_), but not AzBio sentences at +5 dB SNR (S_0_N_0_), when benefit was expressed as acoustic benefit, in rau. However, when controlling for four-frequency PTA and accounting for multiple comparisons, only the relationship between FFR F0 amplitude and acoustic benefit for CNC words remained significant. When bimodal benefit was expressed as normalized acoustic benefit accounting for CI-alone performance, FFR F0 amplitude was only correlated with CNC normalized acoustic benefit. However, that relationship was no longer significant when accounting for multiple comparisons. FFR F1 amplitude was not correlated with any speech recognition measure used in this study.

#### Questionnaire

Eleven of the 14 participants completed the OMSI. Of those who completed the online music survey, the average score was 178.2 (*SD* = 157.8). None of the participants scored over 500 (i.e., classified as more musically sophisticated), meaning all participants were classified as less musically sophisticated. Correlation analyses were completed comparing OMSI scores and FFR spectrum amplitude at F0 and F1 as well as OMSI and acoustic and normalized acoustic benefit for all three speech recognition measures. There were no significant correlations between OMSI scores and FFR F0 or F1 amplitude (*p* > .05).

## Discussion

This study investigated the relationship between bimodal benefit and various measures of behavioral and objective auditory function of the nonimplanted ear. Prior studies have shown little-to-no correlation between measures such as audiometric thresholds in the nonimplanted ear, frequency resolution at a single frequency (i.e., 500 Hz), and temporal envelope resolution with bimodal benefit (Gantz et al., 2009; Gifford, Dorman, Spahr, et al., 2007; Illg et al., 2014; Zhang et al., 2013). SMD thresholds in the nonimplanted ear, on the other hand, have been shown to correlate strongly with bimodal benefit (Zhang et al., 2013). This study sought to replicate these previous studies as well as investigate the relationship between bimodal benefit and an objective measure of auditory function, the FFR, in the nonimplanted ear.

On average, participants in this study demonstrated significant bimodal benefit for all speech measures tested; however, considerable variability was observed consistent with previous studies (Gifford & Dorman, 2019; Mok et al., 2006; Neuman et al., 2017; Zhang et al., 2010). In contrast to previous experiments, we did not observe a relationship between audiometric thresholds (threshold at 125 Hz or four-frequency PTA) and acoustic or normalized acoustic benefit after adjusting for multiple comparisons (Gantz et al., 2009; Illg et al., 2014; Zhang et al., 2013). However, prior studies have shown that the participants with profound low-frequency hearing loss (i.e., no usable hearing) largely drive the correlation between audiometric thresholds and bimodal benefit. Audiometric thresholds are less useful for predicting bimodal benefit for those with moderate-to-severe hearing loss. All of the participants in this study had usable low-frequency hearing in the nonimplanted ear. Given that no participants in this study had audiometric thresholds at 125 Hz exceeding 65 dB HL (mean = 37.5 dB HL, *SD* = 18.6, range = 15 to 65 dB HL; see Figure 1), it is not surprising that we do not see statistically significant relationships between audiometric thresholds and bimodal benefit across all speech recognition measures.

### Spectral Resolution in the Nonimplanted Ear Does Not Significantly Correlate With Bimodal Benefit

Spectral resolution measured via QSMD was not significantly correlated with acoustic or normalized acoustic benefit for any of the speech recognition measures. Thus, these results did not agree with our prediction that QSMD performance would correlate with bimodal benefit. However, many of the participants in this study were fairly high performers on the QSMD task, likely due to the relatively better hearing observed in this sample as compared to previous publications investigating bimodal benefit (Blamey et al., 2015; Illg et al., 2014; Marsh et al., 1993; Zhang et al., 2013). Perhaps a larger sample with a broader range in hearing losses and QSMD performance would reveal a significant relationship between QSMD and bimodal benefit, particularly given the strength of the correlations observed here. Still, it is unlikely that the relationship would be as strong as previously reported (Zhang et al., 2013). Even among the highest QSMD performers, there was considerable variability in bimodal benefit.

One difference between this study and Zhang et al. (2013), who found a significant relationship between SMD and bimodal benefit, was the point on the psychometric function defining threshold for SMD performance. In this article, SMD threshold was defined as 70% correct, whereas Zhang et al. (2013) defined threshold at 79.4% correct. To ensure this difference in threshold was not the cause of this discrepancy between data sets, correlation analyses between QSMD threshold at 79.4% and acoustic and normalized acoustic benefit for each speech recognition measure were also completed. Across all six correlations, there was still no statistically significant relationship between QSMD threshold at 79.4% and acoustic or normalized acoustic benefit for the three speech recognition measures. As was seen with the QSMD threshold of 70%, many of the correlation coefficients would be consistent with a medium or moderate effect size and thus trending toward greater bimodal benefit with better spectral resolution. These results confirm that there is likely a relationship between SMD performance and bimodal benefit; however, this relationship is relatively weak and likely not sufficient for guiding clinical recommendations regarding expected bimodal benefit.

Spectral resolution at 262 Hz and 440 Hz via swept PTC was not significantly correlated with acoustic or normalized acoustic bimodal benefit. These data are consistent with previous research (Gifford, Dorman, Spahr, et al., 2007) and our associated hypothesis. It is possible that spectral resolution at a lower frequency more closely approximating the fundamental frequency of the male talkers for CNC words (123 Hz) and AzBio sentences (131 Hz male talker and 205 Hz female talker) would provide a better relationship between bimodal benefit and frequency resolution—particularly if segregation is playing a critical role in bimodal benefit.

### Temporal Resolution in the Nonimplanted Ear Does Not Significantly Correlate With Bimodal Benefit

Temporal resolution via sinusoidal AM detection was not significantly correlated with acoustic or normalized acoustic benefit for any of the speech recognition measures tested. These results, which match our original hypothesis, replicate findings from Gifford, Dorman, Spahr, et al. (2007) who found no relationship between temporal resolution and bimodal benefit. Temporal resolution for individuals with cochlear hearing loss has been shown to be only slightly worse than that of normal-hearing listeners at equal SLs (Fitzgibbons & Wightman, 1982; Glasberg, Moore, & Bacon, 1987; Nelson & Thomas, 1997). Given our use of low-frequency modulation frequencies, the suprathreshold fixed stimulus presentation level of 90 dB SPL was likely sufficient for near-normal temporal resolution results. We would thus not expect this nearly normal temporal processing at sufficient presentation levels to explain the observed differences in bimodal benefit.

### Relationship Between FFR and Bimodal Benefit

The major finding of this study was the significant positive correlation between FFR F0 amplitude and acoustic benefit for CNC words and AzBio sentences at +5 dB SNR (S_0_N_45–315_) speech recognition tasks even when controlling for four-frequency PTA. The relationship between acoustic benefit for CNC words and FFR F0 amplitude, but not AzBio sentences at +5 dB SNR (S_0_N_45–315_), remained significant when accounting for multiple comparisons. As the strength of the neural representation of F0 increased, acoustic benefit also increased. This trend was also seen for AzBio sentences at +5 dB SNR (S_0_N_0_); however, the relationship did not reach statistical significance (*r* = .52, *p* = .08). It should be noted that this relationship was not consistently seen when bimodal benefit was calculated as normalized acoustic benefit. For normalized acoustic benefit, a significant correlation was only observed with CNC words when controlling for four-frequency PTA but was no longer significant when accounting for multiple comparisons.

One potential explanation is that using normalized acoustic benefit results in much greater variability than acoustic benefit. This greater variability is particularly seen when CI-alone scores approach ceiling or floor performance. The more that CI-alone scores increase, the less acoustic benefit can be derived. Conversely, normalized acoustic benefit considers CI-alone performance, allowing for the full range of benefit (−100 to 100%) to be achieved. Depending on how benefit is calculated, it can appear that the participant receives meager or great bimodal benefit. Although the strength of calculating normalized acoustic benefit is its ability to control for CI-alone performance, perhaps the speech recognition performance of this particular sample lends itself to be better represented by acoustic benefit. No participant reached absolute ceiling effects with the CI-alone or in the bimodal listening condition.

It is also important to note that the relationship between FFR F0 spectral amplitude and bimodal benefit was much stronger for CNC words in quiet compared to both speech recognition measures using AzBio sentences in noise. We suspect that the spectral characteristics of the different speech recognition measures may explain the differences in correlation strength. Zhang et al. (2010) extracted the F0 from one 50-word CNC list and found a mean F0 equal to 123 Hz with a *SD* of 17 Hz, close to the F0 observed of the/da/FFR stimulus. AzBio sentences, on the other hand, use two male and two female speakers. Zhang et al. extracted F0 from 80 sentences and found an average F0 of 131 Hz (*SD* = 35 Hz) and 205 Hz (*SD* = 45 Hz) for male and female speakers, respectively. It is possible that FFR F0 spectral amplitudes better correlated with bimodal benefit for CNC words because the average F0 of CNC words more closely matched the FFR stimulus F0 than AzBio sentences.

To test this, further analysis was completed looking at bimodal benefit for male versus female AzBio sentence recognition at +5 dB SNR (S_0_N_45–315_) and FFR F0 spectral amplitudes. This analysis was only completed for the S_0_N_45–315_ testing condition as male and female percent correct scores are not calculated in the clinical method of AzBio testing at +5 dB SNR (S_0_N_o_). If a speaker F0 that more closely matches the FFR stimulus F0 results in a stronger relationship between FFR spectral amplitudes and bimodal benefit, we would expect to see a stronger correlation between bimodal benefit for male spoken AzBio sentences and FFR F0 spectral amplitudes. Of the 12 participants who had useable FFR data, scores for male and female speakers were available for 11 participants. There was a significant relationship between FFR F0 spectral amplitudes and acoustic benefit for male-spoken (*r* = .71, *p* = .02) and female-spoken (*r* = .77, *p* = .006). These correlations do not differ greatly from each other and closely approximate correlations of acoustic benefit for gender nonspecific sentences as discussed earlier in the article. There was however a greater difference between male- and female-spoken sentences when looking at normalized acoustic benefit. Although neither reached statistical significance, correlations between FFR F0 spectral amplitudes and normalized acoustic benefit for male-spoken AzBio sentences at +5 dB SNR (S_0_N_45–315_; *r* = .42, *p* = .2) were considerably stronger than for female-spoken sentences (*r* = .16, *p* = .63). Although this does not provide sufficient evidence to explain the differences in correlations between CNC and AzBio bimodal benefit with FFR spectral amplitudes, it raises the notion that spectral characteristics of chosen speech stimuli may impact findings. These results highlight the importance of F0 processing for speech recognition and provide additional support to the theory of segregation as an explanation for bimodal benefit.

Interestingly, there was no significant relationship between FFR F1 amplitude and bimodal benefit for any of the speech recognition tasks. We hypothesized that this representation of TFS at F1 would be a key determinant on who would receive bimodal benefit. This lack of a relationship is most likely due to the fact that neural representation of F1 was largely absent for the majority of participants enrolled in this study (see arrow on Figure 4(d)). The first formant of the/da/stimulus was approximately 700 Hz, which likely exceeded the range of frequencies that could adequately be represented in the FFR for our participants with large amounts of hearing loss (see Figure 1). Of the 12 participants included in the FFR analyses, half had audiometric thresholds at 750 Hz of 70 dB HL or greater, effectively reducing or even eliminating, in some cases, audibility for F1 of the stimulus. Furthermore, given our inability to test for dead regions exactly at 700 Hz with the TEN HL test, it is possible that the two participants with dead regions at 750 Hz also had a cochlear dead region at 700 Hz. We would thus not expect to see neural representation at F1 for these participants.

Unfortunately, very few studies have investigated the role that audibility plays on neural representation of F1. In a group of nine participants with mild-to-moderate hearing loss, Ananthakrishnan et al. (2016) found that although participants had neural representation for F1 of a steady-state English back vowel/u/, spectral amplitude did not increase with an increase in SL, unlike NH listeners. However, participants were not tested at SLs below 50 dB SL, which greatly exceeds the SLs for the participants in this study. It is not yet clear how an SL of 10 to 20 dB SL, as was the case for some of the participants in this study, impacts neural representation of F1.

Finally, given the observed relationship between FFR amplitude at F0 and bimodal benefit, but the lack of relationship between temporal resolution and bimodal benefit, further analyses were completed to determine whether there was a relationship between FFR amplitude and temporal resolution measurements. This was assessed as previous study has shown significant relationships between human EFRs and behavioral measures of temporal resolution, including AM detection thresholds, suggesting EFRs reflect similar temporal processing as noted for behavioral tasks (Purcell, John, Schneider, & Picton, 2004). No relationship between AM detection thresholds and FFR F0 amplitude was observed (*p* > .05), regardless of whether participants whose thresholds were assigned a value of 0 were included or excluded. This provides evidence that FFR and AM detection thresholds do not reflect the same type of temporal processing.

### Clinical Implications

The results of this study warrant further investigation to better understand the relationship between FFR amplitude and bimodal benefit. The current findings suggest that the FFR has the potential to be an objective tool that can assess the integrity of the auditory system and help predict bimodal benefit from the nonimplanted ear. It is possible that this information may eventually be used for clinical decision-making, particularly in difficult-to-test populations. Conversely, other measures of auditory function of the nonimplanted ear including audiometric thresholds, spectral resolution, and temporal resolution are not appropriate measures for predicting bimodal benefit. Currently, we lack the tools to provide data-driven counseling regarding the expected amount of bimodal benefit which holds significant clinical implications for optimizing bimodal hearing and determining bilateral CI candidacy—particularly given the poor relationship between audiometric thresholds and bimodal benefit for individuals with moderate-to-severe hearing losses. Without behavioral testing with and without the HA, the decision of whether to pursue a second CI or continue with a bimodal listening configuration is exceedingly difficult. For pediatric patients, by the time such behavioral testing can be completed, it may be too late for optimal speech recognition benefit to be achieved with a second CI if testing shows that the child does not receive benefit from their HA. Further complicating this matter is that some pediatric bimodal listeners exhibit significant asymmetry in neural maturation (Polonenko, Papsin, & Gordon, 2018a, 2019) that may not resolve following receipt of a second CI (Polonenko, Papsin, & Gordon, 2018b). Thus, the FFR may eventually serve as a tool to predict bimodal benefit where behavioral testing is not possible (e.g., pediatric patients and patients with multiple disabilities) affording evidence-based determination of bilateral candidacy.

### Limitations

There are limitations of this study that should be acknowledged. First, age was not controlled. Previous reports have shown a decrease in FFR amplitudes with age in people with NH (Clinard et al., 2010). It is possible that the significant correlation between FFR amplitudes and bimodal benefit was mediated by the age of the participants. However, there was no correlation in the current data between age at testing and the envelope spectrum amplitude at F0 (*r* = .13, *p* = .69) or the fine structure spectrum amplitude at F1 (*r* = .28, *p* = .38). This suggests that age was not mediating the observed relationship between FFR F0 amplitude and bimodal benefit.

Another limitation was the fixed stimulus level used during FFR testing. By fixing the presentation level, it remains unclear whether differences in stimulus SL impacted the results. A fixed level of 90 dB SPL was chosen for this experiment to approximate amplification provided by a HA using the half-gain rule. However, to tease apart the effects of audibility of the stimulus on FFR strength, future studies should present stimuli at a variety of levels and use the participant’s PTA to determine the SL of the stimulus. This will allow for comparisons across fixed stimulus levels as well as across SLs. In this study, however, acoustic benefit for CNC words was still significantly correlated to FFR F0 amplitude even when controlling for four-frequency PTA. Although differences in audibility of the stimulus do influence the observed relationships between FFR F0 and bimodal benefit, they do not fully account for this relationship.

## Conclusion

In conclusion, this study demonstrated a strong positive correlation between FFR F0 amplitude in the nonimplanted ear and bimodal benefit. Other measures of auditory function of the nonimplanted ear such as SMD, audiometric thresholds, and temporal modulation detection were not significantly correlated with bimodal benefit. Further study on the impact of age and audibility on FFR strength must be completed to better understand the relationship between this electrophysiological measure and bimodal benefit.
